# Effect of Clozapine on Anti-N-Methyl-D-Aspartate Receptor Encephalitis With Psychiatric Symptoms: A Series of Three Cases

**DOI:** 10.3389/fnins.2019.00315

**Published:** 2019-04-09

**Authors:** Ping Yang, Liang Li, Shuaishuai Xia, Bin Zhou, Yong Zhu, Gaoya Zhou, Erwen Tu, Tianhao Huang, Huiyong Huang, Feng Li

**Affiliations:** ^1^Department of Psychiatry, Hunan Brain Hospital, Clinical Medical School, Hunan University of Chinese Medicine, Changsha, China; ^2^Provincial Key Laboratory of TCM Diagnostics, Hunan University of Chinese Medicine, Changsha, China; ^3^Shanghai Institute of Measurement and Testing Technology, Shanghai, China; ^4^School of Dentistry, University of California, Los Angeles, Los Angeles, CA, United States

**Keywords:** clozapine, anti-NMDA receptor encephalitis, psychiatric symptoms, antipsychotic therapy, intravenous immunoglobulin, hormone

## Abstract

The main clinical manifestations of anti-N-methyl-D-aspartate receptor (anti-NMDAR) encephalitis are acute or subacute seizures, cognition impairment, and psychiatric symptoms. Nowadays, the scheme of antipsychotic therapy for this disease has not been established. This study reports three cases of anti-NMDAR encephalitis with psychiatric symptoms. The anti-NMDAR antibodies in cerebrospinal fluid (CSF) and serum were positive. The psychiatric symptoms still existed after intravenous immunoglobulin (IVIG) treatment; thus, clozapine was used for antipsychotic therapy. Case 1 was a 37-year-old man who suffered from bad mood and suicide behaviors for 1 month. Hallucination and delusion still existed after IVIG treatment and hormone therapy, and the symptoms were relieved when given clozapine for 12 months. Case 2 was a 28-year-old man who was admitted to our hospital due to injuring other people and destructive behaviors for 2 days. He showed irritability, bad temper, declined cognition, and severe delusion of persecution after IVIG treatment and hormone therapy, but the psychiatric symptoms disappeared when given clozapine for 3 months. Case 3 was a 23-year-old man who suffered from headache and babbing for 7 days. Symptoms such as irritability, bad temper, babbing, and injuring other people still existed after IVIG treatment and hormone therapy, but they disappeared when given clozapine for 2 months. Therefore, we suggest that during the treatment of anti-NMDAR encephalitis with psychiatric symptoms, if the anti-NMDAR antibodies in CSF and serum were positive, and psychiatric symptoms could not be controlled after IVIG and hormone therapy, clozapine may work.

## Introduction

Anti-N-methyl-D-aspartate receptor (anti-NMDAR) encephalitis is an autoimmune encephalitis induced by anti-NMDAR (Jiang et al., 2018). Dalmau et al. (2007) reported anti-NMDAR as the pathogenic antibody and diagnostic marker of this disease in 2007. Since then, the number of newly diagnosed cases has increased year by year (Beecher et al., 2018). At present, anti-NMDAR encephalitis has become a representative in the disease spectrum of autoimmune encephalitis. The number of newly diagnosed cases of anti-NMDAR encephalitis has exceeded that of enterovirus encephalitis and herpes simplex encephalitis (Gable et al., 2012). It often combined with psychiatric symptoms, such as severe hallucination, delusion, and aggressive behaviors (Warren et al., 2018). However, there is no standard treatment for encephalitis with psychiatric symptoms, which brings serious risks and burdens to society and families. This study reported three cases of anti-NMDAR encephalitis with psychiatric symptoms. The anti-NMDAR antibodies were positive in their cerebrospinal fluid (CSF) and blood. All of them were treated with clozapine in our hospital.

## Case Report

### Case 1

**FIGURE 1:**
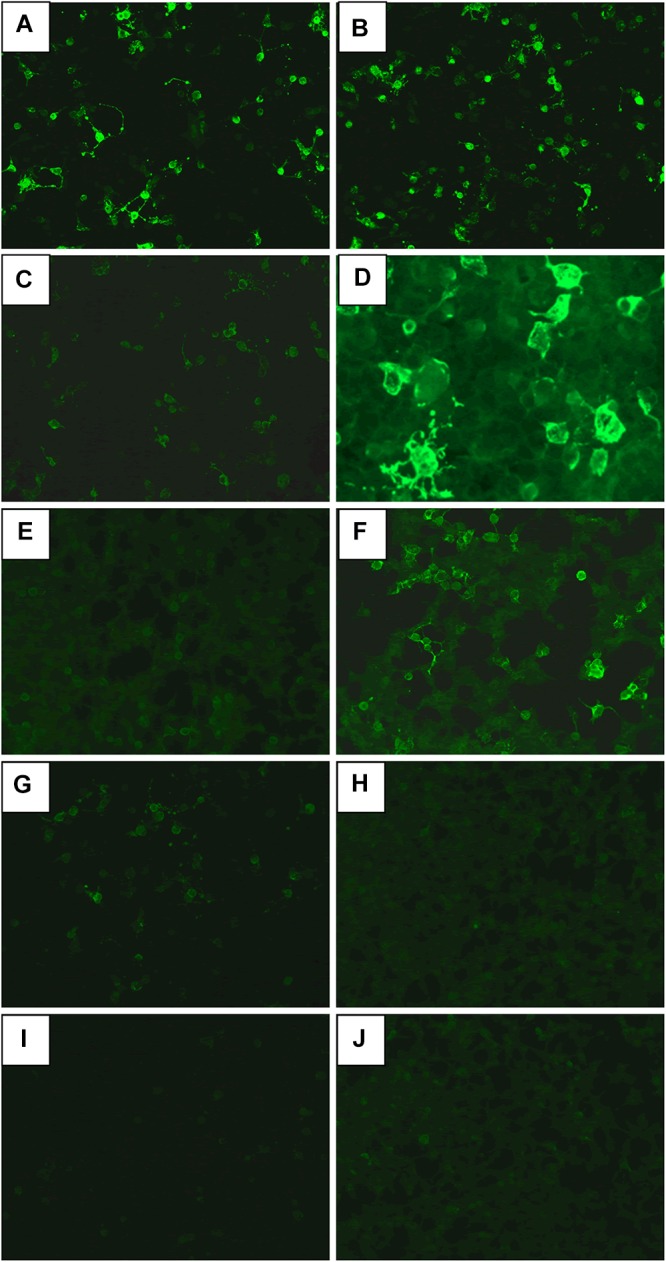
The anti-NMDA receptor (anti-NMDAR) antibodies in CSF **(A,C,E,G,I)** and serum **(B,D,F,H,J)**. **(A,B)** Initial day in case 1. **(C,D)** Before treatment of clozapine in case 1. **(E,F)** After 18-month treatment of clozapine in case 1. **(G,H)** Before treatment of clozapine in case 2. **(I,J)** Before treatment of clozapine in case 3.

**FIGURE 2:**
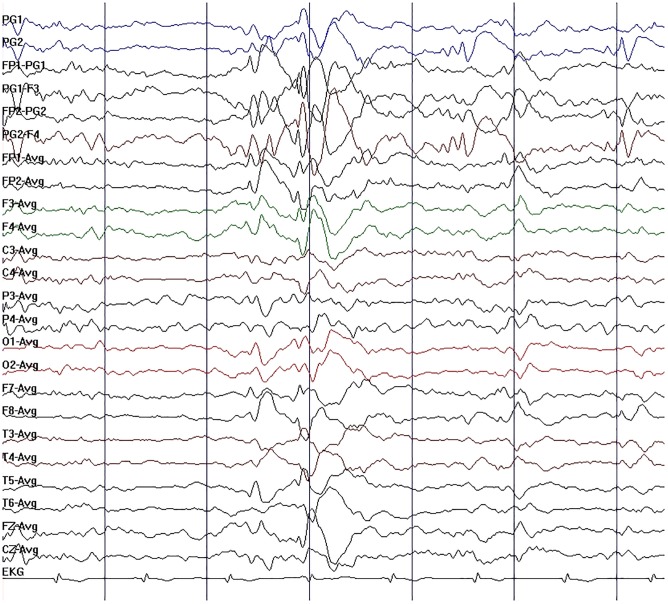
Initial electroencephalography (EEG) in case 1. EEG showed moderate slow-wave discharge, with medium-wave amplitude, appearing as a single emission or continuous occurrence in right sphenoidal electrode, frontal pole, frontal, and pretemporal regions.

**FIGURE 3:**
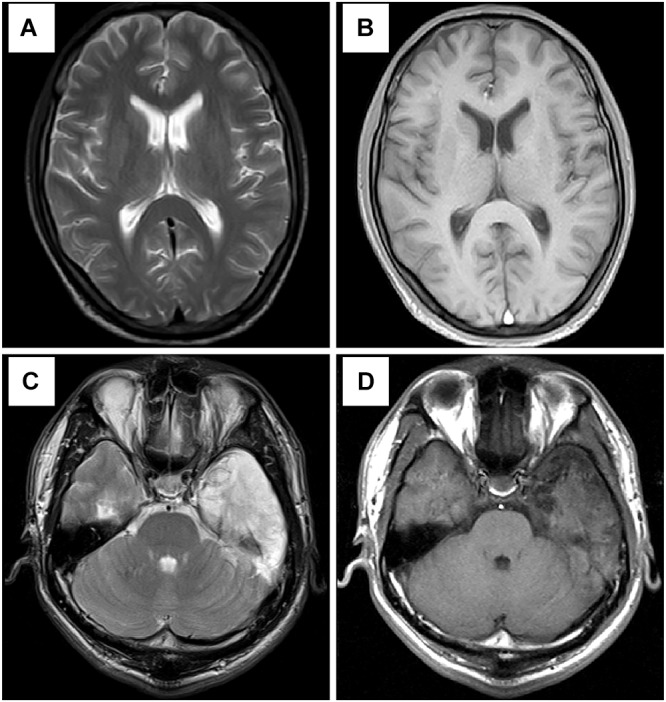
Results of head MRI. **(A,B)** Head MRI showed encephalatrophy in case 1. **(C,D)** Head MRI showed long T1 and long T2 signal intensities in the left temporal lobe, and enhanced MRI stated irregular light enhancement in case 2. **(A,C)** T2WI; **(B,D)** T1-weighted sequence after gadolinium enhancement.

**FIGURE 4:**
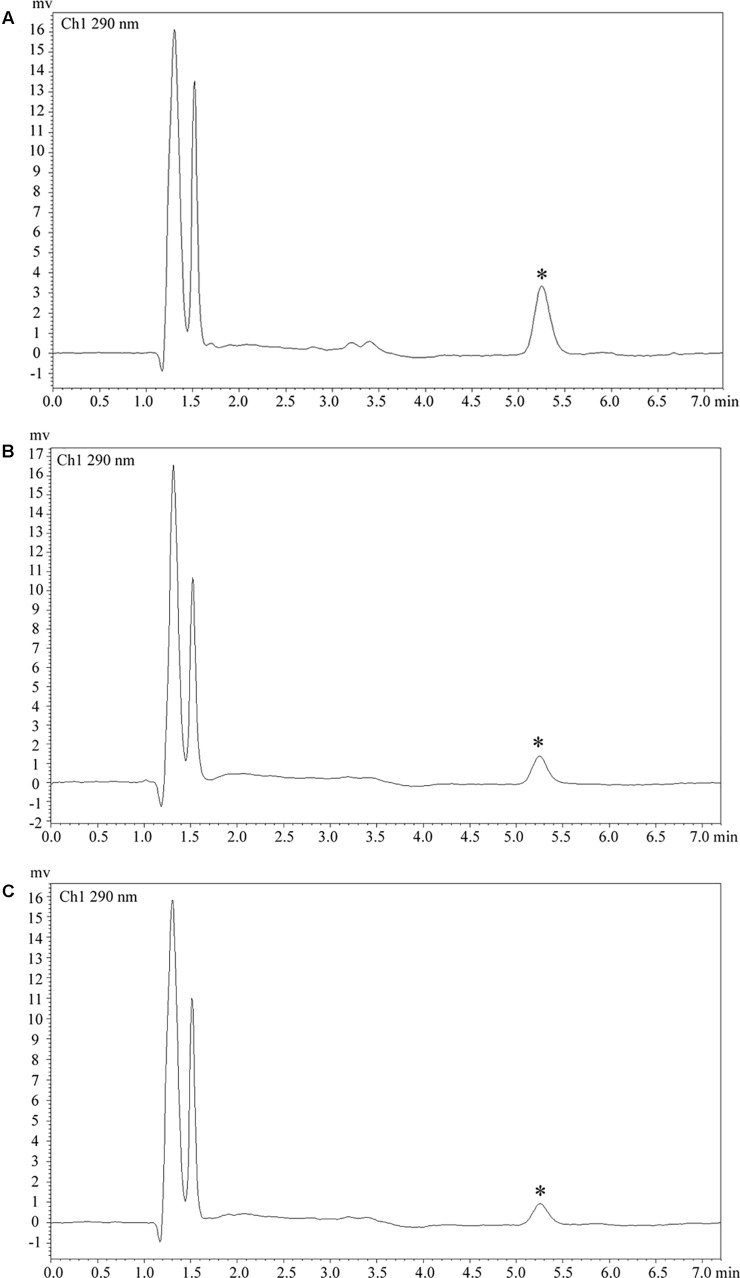
Clozapine plasma concentration. **(A)** Plasma concentration was 218.8 ng/ml in case 1. **(B)** Plasma concentration was 90.6 ng/ml in case 2. **(C)** Plasma concentration was 65.3 ng/ml in case 3. Asterisks (^∗^) represent the chromatogram peak of clozapine.

A 37-year-old male peasant presented with a 4-week history of low spirit, bad mood, suicide behaviors, and suspicion prior to hospitalization. He was diagnosed with severe depression and received sertraline (50–100 mg) and olanzapine (10 mg), but the situation became worse with declined cognition function and epileptic seizures after 7 days of treatment. The CSF pressure was 240 cmH_2_O and leukocyte count was 10 × 10^6^/L. The anti-NMDAR antibodies in CSF and serum were 1:32 (Figure 1A,B). Initial electroencephalography (EEG) showed epileptic activity with sharp-slow waves in the right anterior frontotemporal region (Figure 2). The chest and abdomen were detected with B-ultrasound and CT to exclude tumor. He received intravenous immunoglobulin (IVIG; 25 g/day, 5 days), methylprednisolone (1,000 mg, 3 days + 500 mg, 3 days), and prednisolone (0–60 mg, 12 weeks) for two courses; levetiracetam (1,500 mg, bid) and valproic acid (500 mg, bid) were used to control epilepsy. The patient showed severe heart failure and respiratory failure, with persistent psychiatric symptoms, such as visual hallucination, auditory hallucination, and delusion. When given olanzapine (10–20 mg/day, 3 days) and aripiprazole (2.5–10 mg/day, 7 days), these psychiatric symptoms could not be alleviated. Aggressive behaviors occurred when given olanzapine; muscle stiffness and slurred speech occurred when given aripiprazole. After cessation of olanzapine and aripiprazole, the use of clonazepam (2 mg, bid) led to clinical improvement. Thus, he was sedated with midazolam (2–4 mg/h, 45 days) during the period he was in the intensive care unit (ICU). The patient received quetiapine (50 mg/day to 0.4 g/day, 30 days) and clonazepam (2–6 mg/day, 35 days) from the ICU, but he still had severe visual hallucination and auditory hallucination after 6 months of treatment. Positive and Negative Syndrome Scale (PANSS) total score (Kay et al., 1987) was 112. The anti-NMDAR antibodies in CSF and serum were 1:10 and 1:320, respectively (Figure 1C,D), and the antibodies against AMPA1, AMPA2, LGI1, CASPR2, and GABAb were negative (Suh-Lailam et al., 2013). Head-enhanced magnetic resonance imaging (MRI) showed encephalatrophy (Figure 3A,B), and no epileptic waves were found in EEG. Then, he was given clozapine (50–300 mg/day), with 218.8 ng/ml plasma concentration (Figure 4A; Zhou et al., 2004). Meanwhile, he was still treated with valproic acid (500 mg, bid) for epilepsy control. Eighteen months later, the anti-NMDAR antibodies in CSF and serum were 1:10 and 1:32 (Figure 1E,F), respectively. Up to now, the patient was able to live and work normally, with stable situation and no psychiatric symptoms. PANSS total score was 26.

### Case 2

A 28-year-old male painter suffered from behavioral changes for 1 week after flu prior to hospitalization to the ICU of the local hospital. Head MRI showed long T1 and long T2 signal intensities in the left temporal lobe, and enhanced MRI showed irregular light enhancement (Figure 3C,D). The anti-NMDAR antibodies in CSF and serum were negative. With the diagnosis of viral encephalitis, the patient received antiviral therapy for 35 days, together with methylprednisolone (1,000 mg, 3 days + 500 mg, 3 days) and prednisolone (35–60 mg, 30 days). Then, he left the hospital. Unfortunately, he was admitted to our hospital 2 days after his discharge due to aggressive behaviors, injuring other people, irritability, and severe delusion of persecution. He was given acyclovir and olanzapine (10–20 mg/day), but the symptoms deteriorated with severe violent behavior and declined cognition function after 7 days of treatment. The CSF pressure was 200 cmH_2_O. Total cell count was 58 × 10^6^/L, and leukocyte count was 38 × 10^6^/L. The anti-NMDAR antibodies in CSF and serum were both 1:10 (Figure 1G,H), and the antibodies against AMPA1, AMPA2, LGI1, CASPR2, and GABAb were negative (Suh-Lailam et al., 2013). The chest and abdomen were detected with B-ultrasound and CT to exclude tumor. After treatment with IVIG (30 g/day, 5 days), methylprednisolone (1,000 mg, 3 days + 500 mg, 3 days), and prednisolone (0–60 mg, 12 weeks), the psychiatric symptoms became worse; even olanzapine (10–20 mg/day, 15 days), quetiapine (25–400 mg/day, 15 days), diazepam (5–10 mg/day, 15 days), and clonazepam (2–6 mg/day, 15 days) did not work. PANSS total score (Kay et al., 1987) was 103. Finally, the patient was given clozapine (25–300 mg/day), with 90.6 ng/ml plasma concentration (Figure 4B; Zhou et al., 2004), and all the psychiatric symptoms disappeared completely 3 months later. The patient was discharged. Followed up for 6 months, all the clinical symptoms disappeared. The anti-NMDAR antibodies in CSF and serum were negative, but no obvious changes could be observed in enhanced head MRI. PANSS total score was 21.

### Case 3

A 23-year-old male student was admitted to the local hospital due to headache, babbing, and aggressive behaviors for 1 week. After 7 days of treatment with penicillin and acyclovir, the symptoms were not relieved and then he was transferred to our hospital. No abnormality was found in enhanced head MRI. The CSF pressure was 100 cmH_2_O. Total cell count and leukocyte count were normal. The protein concentration was 0.46 g/L. The anti-NMDAR antibodies in CSF and serum were 1:1 and 1:10, respectively (Figure 1I,J), and the antibodies against AMPA1, AMPA2, LGI1, CASPR2, and GABAb were negative (Suh-Lailam et al., 2013). The chest and abdomen were detected with B-ultrasound and CT to exclude tumor. PANSS total score (Kay et al., 1987) was 97. After treatment with IVIG (25 g/day, 5 days), methylprednisolone (1,000 mg, 3 days + 500 mg, 3 days), and prednisolone (0–60 mg, 12 weeks), followed by antipsychotic therapy with olanzapine (10–20 mg/day, 15 days), quetiapine (25–400 mg/day, 15 days), and clonazepam (2–4 mg/day, 30 days), the patient still showed visual hallucination and aggressive behaviors. Then, he was given clozapine (50–100 mg/day), with 65.3 ng/ml plasma concentration (Figure 4C; Zhou et al., 2004). The psychiatric symptoms disappeared after 2 months of treatment. Followed up for 6 months, he was able to live and work normally. The anti-NMDAR antibodies in CSF and serum were negative. PANSS total score was 18.

## Discussion

The incidence of anti-NMDAR encephalitis is second only to acute disseminated encephalomyelitis in autoimmune encephalitis (Granerod et al., 2010). Anti-NMDAR encephalitis may initially present with multiple psychiatric symptoms, which results in being misdiagnosed as primary psychiatric disease (Dalmau et al., 2008).

A study showed that among 111 anti-NMDAR encephalitis patients, 65 (58.6%) presented various psychiatric features, 43 (38.7%) were admitted initially to a psychiatric unit, and 2 (1.8%) were transferred from other inpatient units to a psychiatric unit before being finally correctly diagnosed (Lejuste et al., 2016). It was reported that catatonia was highly suggestive of NMDAR encephalitis, helping to diagnose anti-NMDAR encephalitis (Mythri and Mathew, 2016). The three patients in this study presented depression and aggressive behaviors, without catatonic syndrome. They were diagnosed with viral encephalitis and primary psychiatric disorder in the early stage, which delayed treatment. Gurrera believed that without appropriate treatment, patients are likely to suffer a protracted course with significant residual disability or death (Gurrera, 2018). At present, there is no formal antipsychotic treatment program for anti-NMDAR encephalitis with psychiatric symptoms. There are only few case reports about this; thus, treatment of such patients becomes more difficult. No specific medicine was found to improve the patient’s psychiatric symptoms. For example, in the two cases reported by Kuppuswamy, olanzapine only worked in one patient, while aggravating the other patient’s mental symptoms (Kuppuswamy et al., 2014). The side effects of some drugs, such as aripiprazole and haloperidol, even worsen the difficulties experienced during treatment (Chapman and Vause, 2011). In case 1, before the use of clozapine, the severe side effects caused by antipsychotics resulted in many extrapyramidal symptoms and serious aggressive behaviors. The dose of the medicine could not be increased gradually as usual, and the treatment had to be interrupted.

It was reported that NMDAR hypofunction was a potential mechanism resulting in schizophrenia, which complemented the most widespread explanatory mechanism of “dopamine hypothesis” for schizophrenia (Ramanathan et al., 2014). Some scholars declared that NMDAR dysfunction was the “final common pathway” underlying the pathogenesis of schizophrenia, and it was associated with both positive and negative symptoms (Wang et al., 2017). Anti-NMDAR antibodies were also found in schizophrenia patients (Koshiyama et al., 2018; Xie and Huang, 2018). Thus, some researchers believed that schizophrenia and anti-NMDAR encephalitis may have the same underlying mechanism and could be on the same spectrum (Maneta and Garcia, 2014). However, there is not enough proof to date to verify whether they are diseases on the same spectrum or under two different conditions.

The NMDAR, an ionotropic glutamate receptor, is related to synaptic plasticity, neuronal maturation, study, and memory (Hanson et al., 2015). NMDARs are heteromers of NMDAR1 and NMDAR2 subunits, which bind with glycine and glutamate, respectively (Tachibana et al., 2013). NMDAR hyperfunction is proposed to result in psychosis (Liu et al., 2015). Anti-NMDAR encephalitis represents a state of NMDAR hypofunction caused by autoantibodies against NMDAR (Warikoo et al., 2018). Thus, antipsychotic drugs affecting glutamate should be chosen during the treatment. Studies have shown that clozapine was the first atypical antipsychotic drug created successfully in the late 1960’s. It is a diphenyldiazepine antipsychotic drug, with strong sedative and hypnotic effects, which can directly inhibit the ascending reticular activating system in the brainstem. It can selectively act on the mesencephalic limbic dopamine and the 5-serotonin (5-HT) systems, as well as the muscarinic and α1-noradrenergic receptor systems. Clozapine blocks the dopamine receptors reversibly and increases dopamine retroconversion. It has strong anticholinergic, antisympathetic, and antihistamine effects (Shi et al., 2007). At present, the pharmacological research based on the glutamatergic hypothesis of schizophrenia can go further among all the most promising mechanisms. It was reported that clozapine can affect 5-HT2A and D4 receptors, increase the release of dopamine, and selectively increase the concentration of Glu in the prefrontal cortex ( Lopez-Munoz and Alamo, 2011). Incrocci et al. (2018) stated that the atypical antipsychotic clozapine potently blocked the disruption of the sensorimotor gating induced by NMDA antagonists. Rebollo et al. (2018) found that decreasing hypersynchronization in the local circuit may be one of the mechanisms of clozapine in preventing schizophrenia symptoms derived from NMDA hypofunction.

In this study, three patients of anti-NMDAR encephalitis with psychiatric symptoms were included. They had aggressive behaviors, even injured other people and destroyed objects. After IVIG treatment and hormone therapy, the use of various antipsychotics, such as midazolam, olanzapine, quetiapine, and aripiprazole, could not alleviate psychiatric symptoms. Olanzapine even aggravated their aggressive behaviors. Some researchers suggested that modern electroconvulsive therapy (MECT) should be used appropriately (Mann et al., 2012; Jones et al., 2015). However, anti-NMDAR encephalitis mainly showed epileptic seizures, status epilepticus, ventilation and air exchange dysfunction, autonomic nervous dysfunction, and multisystem complications. The situation was so serious that the patients had to be treated with intubation and auxiliary ventilation in the ICU. Therefore, MECT was not suitable for them. Furthermore, severe psychiatric symptoms prevented the patients from completing intensive care. In case 1 of this study, intensive care was interrupted due to severe visual and auditory hallucinations. With the improvement of seizure control, ventilation, and air exchange dysfunction, clozapine (300 mg) was used, and the psychiatric symptoms completely disappeared after 1 year of treatment. The patients in cases 2 and 3 showed violence and serious injuring tendency. The aggressive behaviors occurred after treatment of olanzapine, and other antipsychotics such as quetiapine and aripiprazole could not control psychiatric symptoms. In the end, they were given clozapine 300 and 100 mg, respectively, which controlled the symptoms well.

To sum up, clozapine can be used in the treatment of anti-NMDAR encephalitis with psychiatric symptoms. The disease should be under the control of epilepsy and good ventilation function; otherwise, clozapine may induce epilepsy (Bolu et al., 2017). It may be the reason why many doctors are reluctant to select clozapine. Therefore, when clozapine is used, it is important to monitor the EEG regularly to assess the risk of epilepsy. These three patients received EEG monitoring every month after taking clozapine, and the results were normal. Furthermore, antiepileptics, such as sodium valproate and levetiracetam, should not be ignored if necessary. Clozapine must be used on the basis of the ineffectiveness of three antipsychotics to anti-NMDAR encephalitis, just like the treatment of refractory schizophrenia. In this study, quetiapine and aripiprazole were ineffective to all the patients, and the psychiatric symptoms worsened after olanzapine. It is speculated that the affinity of receptor subtypes is different, and the exact mechanism needs further study.

## Conclusion

We report three cases of anti-NMDAR encephalitis with psychiatric symptoms. During the treatment of the disease, if the psychiatric symptoms could not be controlled after IVIG and hormone therapy, clozapine may work.

## Ethics Statement

This study was approved by the Ethics Committee of the Hunan Brain Hospital. A written informed consent was obtained from the patients for the publication of this case report.

## AUTHOR CONTRIBUTIONS

PY and LL conceived the idea, revised all the literature, and wrote the manuscript. SX, YZ, and GZ collected the clinical data. BZ and TH analyzed and interpreted the head MRI. ET performed and analyzed the EEG. HH and FL contributed to the revision of the manuscript and read and approved the submitted version.

## Conflict of Interest Statement

The authors declare that the research was conducted in the absence of any commercial or financial relationships that could be construed as a potential conflict of interest.
